# Care Coordination Can Reduce Unmet Needs of Persons With Severe and Persistent Mental Illness

**DOI:** 10.3389/fpsyt.2019.00563

**Published:** 2019-08-09

**Authors:** Anton Isaacs, Alison Beauchamp, Keith Sutton, Nilay Kocaali

**Affiliations:** ^1^School of Rural Health, Monash University, Traralgon, VIC, Australia; ^2^Department of Rural Health, Monash University, Warragul, VIC, Australia; ^3^Gippsland Primary Health Network, Traralgon, VIC, Australia

**Keywords:** needs assessment, psychiatric rehabilitation, severe mental disorders, care coordination, accommodation, housing, community mental health services, mental health services

## Abstract

**Introduction:** Persons with severe and persistent mental illness (SPMI) have multiple and complex needs, many of which are not health related. Mental health services are unable to address these needs without collaboration with other agencies. In the absence of this collaboration, persons with SPMI often fall through the system cracks and are unlikely to experience recovery. Furthermore, previous studies have shown that unmet accommodation needs are associated with unmet needs in other areas. This study aimed to ascertain whether a care coordination model adopted in Australia’s Partners in Recovery [PIR] initiative was able to reduce unmet needs in such persons and also if meeting accommodation needs were associated with meeting other needs.

**Methods:** This was a longitudinal study where met and unmet needs of clients measured using the Camberwell Assessment of Needs Short Appraisal Schedule (CANSAS) were compared at enrolment and exit from the PIR initiative. Logistic regression was used to examine the association between change in accommodation needs and change in other CANSAS variables.

**Results:** In total, 337 clients (66% of 508 clients) had both baseline and follow-up data and were seen within the time frame of 14 to 101 weeks. At baseline, the most frequently reported unmet needs were psychological distress, daytime activity, and company (89%, 72%, and 67%, respectively). At follow-up, these had decreased to 27%, 22%, and 22%, respectively. The proportions of clients with an unmet need at baseline who subsequently progressed to having that need met at follow-up ranged between 62% and over 90%. Change in accommodation needs from unmet to met was associated with changes in monetary needs and needs related to childcare, food, safety to self, education, and access to other services, with the greatest change seen for monetary needs (adjusted OR 2.87, 95% CI 1.76, 4.69).

**Conclusions:** Reducing needs of persons with SPMI is the starting point of recovery and is a good indicator of psychiatric care. Care coordination is a useful way to address multiple and complex needs of persons with SPMI. While addressing needs, priority must be given to meeting accommodation needs.

## Introduction

Persons with severe and persistent mental illness (SPMI) have multiple and complex needs, which are generally beyond the scope of traditional mental health services (1). Assessment of need is a good general measure of the number and severity of a client’s problems in everyday life (2) and is critical in mental health rehabilitation (3). Hence, a change in needs from unmet to met gives an indication of the effectiveness of psychiatric care (4). People with a number of unmet needs are likely to experience a poor quality of life (5), and the longer these needs remain unmet, the less are the chances of recovery. However, when these needs are met, recovery becomes easier (6). There is hence a need for a reorganization of care delivery for people with multiple needs that focuses on recovery by addressing client needs and better care coordination (7). Any mental health service that aims to improve the quality of life of their clients, needs to actively assess and address their reported needs (6). The recognition of factors associated with each unmet need can help optimize planning and implementation of care (8).

The most common unmet needs reported by persons with SPMI are psychological distress, help with psychotic symptoms, daily activities, company/someone to spend time with, employment and volunteering, physical health problems, and those relating to money (1, 9, 10). A recent report has suggested that when accommodation needs are unmet, several other needs remain unmet (9). This observation is in accordance with previous research. For instance, people who have a mental illness and are homeless are less likely to receive public benefits and are more likely to experience severe poverty (11–13). Poverty is also the underlying cause of homelessness (14).

Homeless people with mental health problems are known to experience food insecurity (15, 16) because homelessness prevents the preparation and storage of food as well as precludes market and nonmarket activities needed for the preparation of food (17). Homeless persons also spend less on food and eat fewer meals than their housed counterparts (18). Homeless people do not have the means to afford transport even for basic needs, such as obtaining food or accessing health services (19). Homeless families also have difficulty caring for their children. They are forced to make big sacrifices for them, protecting them from harm and struggling with the restrictions of not having a home (20).

Medical problems are particularly prevalent among homeless people. Seizures, chronic obstructive pulmonary disease, (21), and oral and dental diseases are common (22). Continuous exposure to the elements predisposes homeless persons to respiratory infections and skin disease. Prolonged exposure to moisture, inadequate footwear, and walking long distances results in foot disorders, such as onychomycosis, tinea pedis, corns, and callouses (23, 24). Moreover, chronic conditions, such as hypertension, diabetes, and anemia are often either not treated or remain undiagnosed (25, 26). Violence is a constant threat to homeless people with high incidences of assault, murder, and rape. Physical injuries caused by falls and being struck by motor vehicles are also common among people who are homeless (25). Homeless people with a mental illness are unable to readily access health care services, which tend to exclude them as a result of stigma, prejudice, and the inadequacy of care available for their complex needs (27, 28).

A recovery-oriented service system that aims to address unmet needs of persons with SPMI requires collaboration between mental health services and other agencies, such as housing, welfare, general practices, and alcohol and drug services. This can be done either by service system integration (29) or by integration at the service delivery level (30, 31). Whereas integrating service systems is plagued with several barriers, such as inability to share information and reluctance of staff to take on more caseloads (29), integration at the service delivery level appears to be more feasible (32).

Care coordination is an example of integration at the service delivery level and has been identified as a core requirement for provision of such care (33, 34). Care coordination involves working with persons with SPMI to first identify and prioritize their needs, then liaising with multiple service providers to develop a care plan, and finally facilitating the provision of services according to that plan to meet clients’ needs (32, 35). Care coordination was originally introduced to mental health services in the United States several decades ago (33), and the role came to be undertaken by the case manager. However, because of an increased workload, case managers in Australia now mostly focus on medication compliance, early warning signs, and crisis management, with little time for recovery-oriented work (35).

The Partners in Recovery (PIR) initiative of the Australian Government was set up to facilitate better coordination between clinical and other supports, to strengthen partnerships, to improve referral pathways, and to promote a community-based recovery model for persons with SPMI (36). It aimed to cover 24,000 people through 48 agencies across the country (36). The initiative was originally implemented from 2012 to 2016 and then extended until mid-2019 to enable transition to the National Disability Insurance Scheme (37).

**Figure 1:**
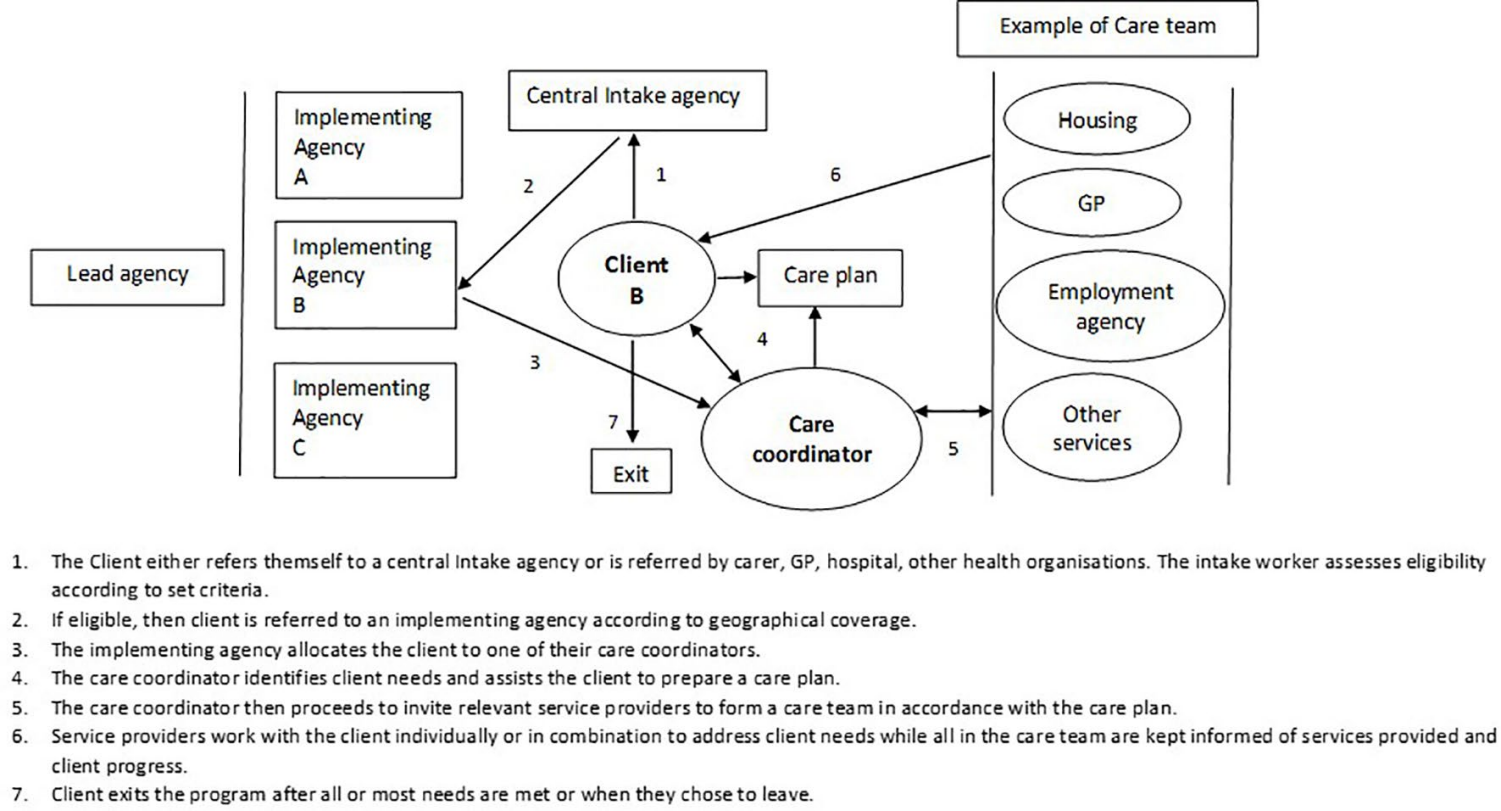
The PIR model of care coordination.

The PIR model involved a regional lead organization that guided and supported implementing organizations. Each implementing organization had a team of care coordinators who worked with clients to develop a care plan based on their needs. Once a care plan was developed, the care coordinator (referred to as a support facilitator in the PIR program) brokered services from relevant agencies in accordance with the plan. Hence, the PIR initiative primarily aimed to reduce unmet needs of clients. Met and unmet needs were documented and monitored regularly during client-care coordinator meetings. Clients exited the program when they chose to or once most of their needs were met. The PIR initiative is described in **Figure 1**.

### Conceptual Framework

To improve one’s quality of life, fulfillment of one’s needs is essential (38).

**Figure 2:**
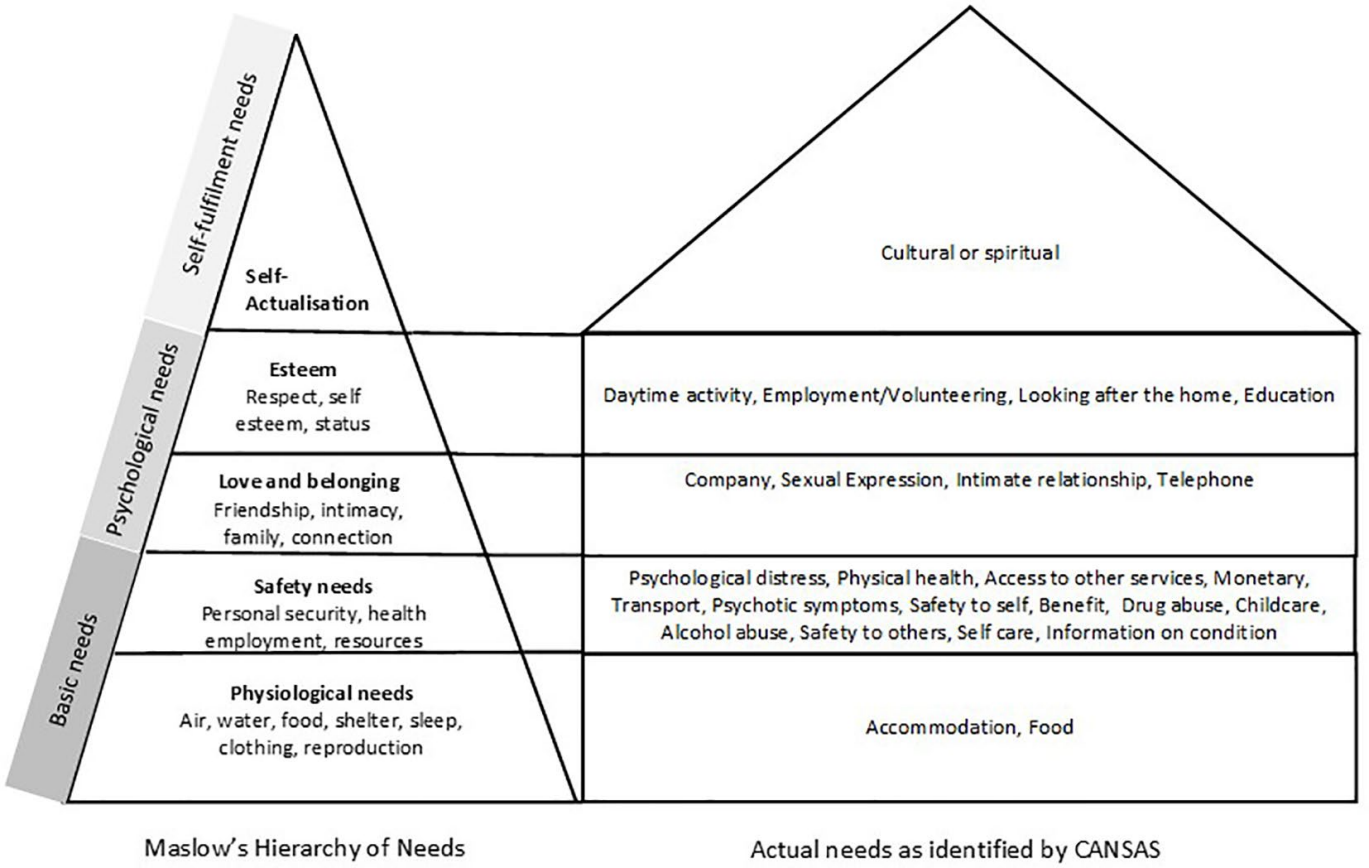
Conceptual framework showing needs in Maslow’s hierarchy and CANSAS.

The conceptual framework developed for this study draws from aligning needs listed by the Camberwell Assessment of Need Short Appraisal Schedule (CANSAS) with Maslow’s hierarchy of needs (39, 40) (see **Figure 2**). When needs listed by the CANSAS are classified according to Maslow’s five-stage hierarchy, accommodation and food needs are basic physiological needs, and people tend to achieve physiological needs before other higher-level needs (38, 41). In the context of persons with SPMI, we postulate that addressing basic needs is necessary to be able to address higher-level needs. If accommodation (housing) needs are met, people will be better placed to receive social benefits, access better quality food, look after and protect their children, stay safe, and more readily access services.

Hence, we proposed two hypotheses:

1. That enrolling in the PIR initiative would reduce the number of clients’ unmet needs; and
2. That meeting accommodation needs would be associated with meeting other higher-level needs.

## Methods

### Study Design

This was a longitudinal study where met and unmet needs of clients were compared at enrollment and exit from the Gippsland PIR initiative.

### Setting

This study was conducted in Gippsland—a non-metropolitan area in the state of Victoria with a population of over 270,000 people and covering an area of 41,600 km^2^ (42). The PIR initiative in Gippsland is overseen by a not-for-profit regional health planning commissioning organization called Gippsland Primary Health Network (PHN) that formed a regional consortium with three Community Mental Health Support Services (CMHSS) and the Area Mental Health Service (AMHS) to implement and govern this initiative. CMHSSs are not-for-profit organizations specialized in recovery-focused nonclinical mental health service delivery. The AMHS adult services include an acute psychiatry inpatient service and a secure extended care unit located at the regional referral hospital, a residential rehabilitation care unit, a prevention and recovery care service, and community mental health teams dispersed across the region. The Gippsland PIR Consortium was later joined by a local Aboriginal Community Controlled Health Organization and the provider of intake services to the Gippsland PIR initiative.

### Data Source

Data on clients who enrolled for the PIR initiative in Gippsland are stored by Gippsland PHN on an online purpose built client information management system called Fixus (43). The Fixus database contains demographic data and scores from CANSAS. The CANSAS is the most commonly used instrument for needs assessment in mental health services (44–46). For the PIR initiative, three additional social and health domains, namely, employment and volunteering, cultural and spiritual, and other services, were added to the original 22 domains (47). Support facilitators verbally obtained and documented client responses on the Fixus database. Deidentified Fixus data were obtained in February 2019. Ethics approval for the study was obtained from Monash University Human Research Ethics Committee (Project ID: 17216; 18/12/2018–18/12/2023).

### Data Analysis

Data were analyzed using Stata 15 (48). Demographic and health status data are reported as proportions or mean and standard deviation. For each area examined using the CANSAS instrument, “no problems (no needs)” and “some problems (needs met)” were coded as “needs met” versus “serious problems” or “unmet needs” (coded as “unmet needs”) and are reported as the proportions of participants with unmet needs at baseline who progressed to having those needs met at follow-up.

Logistic regression was used to examine the association between change in accommodation needs as the independent variable and change in other CANSAS variables as the dependent variables. In this analysis, “change” was defined as moving from having an unmet need at baseline to having a met need at follow-up. Odds ratios (ORs) and 95% confidence intervals (CIs) described the likelihood of changing from an unmet need to a met need in one area if accommodation needs also changed. The model was adjusted for age, sex, and time between baseline and follow-up (“weeks”). These confounders were included because they were intermittently associated with either unmet accommodation needs or other unmet needs using logistic regression. The variable “weeks” was not normally distributed and was transformed for analysis purposes. There was also a wide variation in this variable, with a range of 1 to 166 weeks. The 10th and 90th percentiles were used as cutoff points, excluding 98 clients from analysis. A further 73 clients were excluded because of having no follow-up data.

## Results

In total, 337 clients (66% of 508 clients) had both baseline and follow-up data and were seen within the time frame of 14 to 101 weeks. Differences in demographic and health characteristics between those included in analysis and those excluded because of time frames or incomplete follow-up were statistically significant for mean age only (included mean age 45.7 years, SD 11.3, excluded mean age 42.2 years, SD 11.1, p < 0.001). No other statistically significant differences between the included and excluded groups were seen.

Of the 337 included clients at baseline (**Table 1**), 56.1% were female, 49.3% lived alone, 48.8% had never married, 57.1% were not in the labor force, and 58.5% had senior secondary education or above. The mean number of weeks between baseline and follow-up was 50.8 weeks (SD 23.6). Most clients lived in a private residence (82.2%), and 46.0% had an accommodation tenure of <1 year (**Table 2**). As shown in **Table 3**, the most frequent principal diagnosis was mood (affective) disorders, whereas general practitioners were the most common service provider (54.3%).

**Table 1:** Baseline demographic data for n = 337 clients with severe and persistent mental illness participating in the Partners in Recovery Initiative with both baseline and follow-up data.

Variable name	Number	Percent
Female	189	56.1%
*Age*		
Mean age in years (standard deviation)	45.7 (11.3)	
< 30 years	31	9.2%
30–39 years	75	22.2%
40–49 years	98	29.1%
50–59 years	93	27.6%
60+ years	40	11.9%
*Living arrangements*		
Couple with child(ren)	25	7.4%
Couple without child(ren)	29	8.6%
Group	18	5.3%
Lone person	166	49.3%
Not or inadequately described	6	1.8%
One parent with child(ren)	41	12.2%
Other family	52	15.4%
*Relationship status*		
Married/registered or *de facto*	48	14.3%
Divorced	58	17.3%
Separated	51	15.2%
Widowed	10	3.0%
Never married	165	48.8%
Not adequately described	5	1.5%
*Employment status*		
Employed	20	6.0%
Unemployed	123	36.6%
Not in labor force	192	57.1%
Not adequately described	1	0.3%
*Educational attainment*		
Postgraduate degree level	5	1.5%
Bachelor degree	13	3.9%
Graduate diploma and graduate certificate level	5	1.5%
Advanced diploma and diploma level	21	6.2%
Certificate level	52	15.4%
Senior secondary education	101	30.0%
Junior secondary education	113	33.5%
Primary education	9	2.7%
Other education	2	0.6%
No education	1	0.3%
Not stated/inadequately described	15	4.5%
*Time between baseline and follow up (weeks)*		
Mean (standard deviation)	50.8 (23.6)	
Minimum–maximum	14–101	

**Table 2:** Accommodation type at baseline for n = 337 clients with severe and persistent mental illness participating in the Partners in Recovery Initiative with both baseline and follow-up data.

Variable name	Number	Percent
Type of accommodation		
Private residence	277	82.2%
Residential aged care service	4	1.2%
Domestic-scale supported living facility	1	0.3%
Other supported accommodation	7	2.1%
Other accommodation, not elsewhere classified	22	6.5%
Specialized alcohol/other drug treatment residence	2	0.6%
Specialized mental health community-based residential support service	6	1.8%
Boarding/rooming house/hostel or hostel-type accommodation	4	1.2%
Shelter/refuge	0	
Homeless persons’ shelter	0	
Public place (homeless)	9	2.7%
Prison/remand center/youth training center	1	0.3%
Psychiatric hospital	0	
Unknown/unable to determine	4	1.2%
Accommodation tenure		
< 1 year	155	46.0%
1–2 years	52	15.4%
3–4 years	43	12.8%
≥5 years	74	22.0%
Not stated/inadequately described	13	3.9%

**Table 3:** Health data at baseline for n = 337 clients with severe and persistent mental illness participating in the Partners in Recovery Initiative with both baseline and follow-up data.

Variable name	Number	Percent
*Principal diagnosis*		
F00–F09 Organic, including symptomatic	11	3.3%
F10–F19 Mental and behavioral disorder	18	5.3%
F20–F29 Schizophrenia, schizotypal and delusional disorders	51	15.1%
F30–F39 Mood (affective) disorders	160	47.5%
F40–F48 Neurotic, stress-related and somatoform disorders	21	6.2%
F50–F59 Behavioral syndromes	10	3.0%
F60–F69 Disorders of adult personality	27	8.0%
F70–F79 Intellectual disability	3	0.9%
F80–F89 Disorders of psychological development	3	0.9%
F90–F98 Behavioral and emotional disorders with childhood onset	18	5.3%
F99–F99 Unspecified mental disorder	15	4.5%
*Main service provider*		
General practitioner	183	54.3%
Public sector mental health service	99	29.4%
Private mental health professional	23	6.8%
Other	9	2.7%
None	10	3.0%
Not stated/unknown	4	1.2%

**Figure 3:**
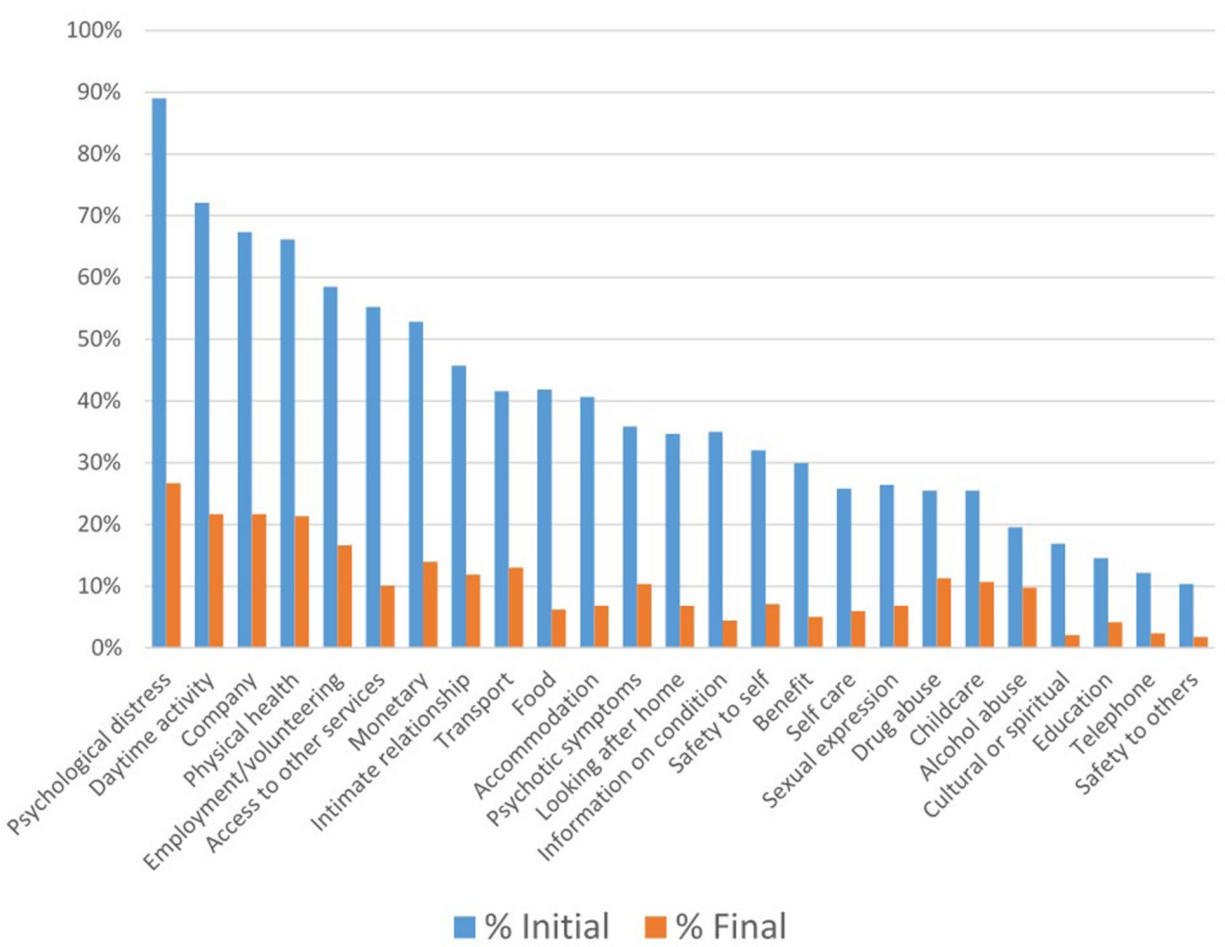
Unmet needs at baseline and follow-up in n=337 clients with severe and persistent mental illness.

The proportion of unmet needs for each variable measured in the CANSAS is shown in **Figure 3**. At baseline, the most frequently reported unmet needs were psychological distress, daytime activity, and company (89%, 72%, and 67%, respectively). At follow-up, these had decreased to 27%, 22%, and 22%, respectively. The least frequently reported unmet needs were basic education, telephone, and safety to others (15%, 12%, and 10%, respectively). At follow-up, these had decreased to 4%, 2%, and 2%, respectively).

**Figure 4:**
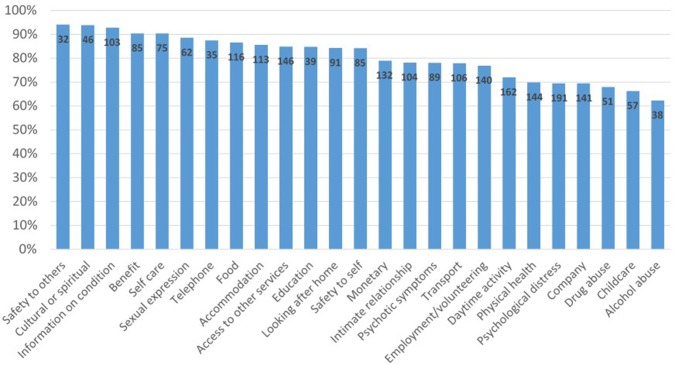
Proportion of clients with an initial unmet need who reported a met need or no problem at final follow-up.

The proportions of clients with an unmet need at baseline who subsequently progressed to having that need met at follow-up ranged between 62% and over 90% (**Figure 4**). The highest proportion of clients showing a change was seen for the variables safety to others (94.1%) and cultural or spiritual needs (93.9%). The lowest proportion was seen for alcohol abuse and childcare needs (62.3% and 66.3%, respectively). **Table 4** shows associations between change in accommodation needs and change in other CANSAS variables between baseline and follow-up. The greatest change was seen for monetary needs (adjusted OR 2.87, 95% CI 1.76, 4.69), whereby the likelihood of changing from unmet to met monetary need was almost three times greater when accommodation needs also changed from unmet to met. Significant associations were also seen between changes in accommodation needs and changes in needs related to childcare, food, safety to self, education, and access to other services.

**Table 4:** Association between change in accommodation needs and change in other needs at final assessment before and after adjustment for covariates.

	Model 1 (unadjusted)		Model 2 (adjusted)*	
*Met need at final assessment*	*OR*	*95% CI*	*OR*	*95% CI*
**1. Monetary needs** (n = 308)	**2.74**	**1.69, 4.44**	**2.87**	**1.76, 4.69**
**2. Childcare needs** (n = 317)	**2.68**	**1.50, 4.81**	**2.90**	**1.58, 5.33**
**3. Food needs** (n = 320)	**2.17**	**1.35, 3.49**	**2.23**	**1.38, 3.61**
**4. Safety to self needs** (n = 294)	**1.96**	**1.17, 3.30**	**1.98**	**1.18, 3.33**
**5. Education needs** (n = 313)	1.94	0.99, 3.82	**2.05**	**1.03, 4.08**
**6. Access to other services** (n = 304)	**1.63**	**1.02, 2.63**	**1.72**	**1.06, 2.80**
7. Transport needs (n = 319)	1.50	0.92, 2.44	1.56	0.95, 2.57
8. Looking after home needs (n = 299)	1.41	0.84, 2.36	1.42	0.85, 2.40
9. Telephone needs (n = 322)	1.36	0.66, 2.80	1.44	0.68, 3.02
10. Physical health needs (n = 307)	1.34	0.84, 2.16	1.34	0.83, 2.16
11. Company needs (n = 297)	1.32	0.81, 2.13	1.31	0.81, 2.12
12. Daytime activity needs (n = 310)	1.32	0.83, 2.12	1.33	0.83, 2.13
13. Psychological distress needs (n = 301)	1.32	0.80, 2.18	1.33	0.81, 2.20
14. Cultural or spiritual needs (n = 277)	1.29	0.67, 2.49	1.37	0.71, 2.68
15. Psychotic symptoms needs (n = 291)	1.28	0.76, 2.14	1.28	0.76, 2.15
16. Self-care needs (n = 310)	1.27	0.74, 2.17	1.28	0.75, 2.20
17. Drug abuse needs (n = 298)	1.25	0.67, 2.33	1.29	0.69, 2.41
18. Safety to others needs (n = 296)	1.20	0.56, 2.57	1.26	0.58, 2.71
19. Information on condition needs (n = 313)	1.19	0.73, 1.95	1.24	0.76, 2.05
20. Employment/volunteering needs (n = 304)	1.17	0.73, 1.87	1.19	0.74, 1.93
21. Alcohol abuse needs (n = 299)	1.13	0.56, 2.87	1.14	0.56, 2.33
22. Sexual expression needs (n = 222)	0.98	0.52, 1.88	0.99	0.51, 1.93
23. Benefit needs (n = 296)	0.85	0.50, 1.47	0.87	0.50, 1.50
24. Intimate relationship needs (n = 274)	0.74	0.44, 1.26	0.77	0.45, 1.34

OR, odds ratios; CI, confidence interval; *Model 2 adjusted for age, sex, and time in weeks between initial and final assessment. Odds ratios and 95% CI in bold are statistically significant.

To test whether associations were similar according to length of time in PIR, we categorized weeks in PIR into three groups. Similar patterns of association were seen for all three categories but were more likely to be significant in categories of longer periods of time.

## Discussion

This study describes a care coordination model that was associated with a reduction in unmet needs of persons with SPMI. As discussed earlier, reducing client reported needs is the starting point of recovery (6, 49) and is a good indicator of psychiatric care (4). Although some needs such as finding a partner can be difficult to assess properly, others might be more difficult to meet, such as needs related to substance abuse. Nonetheless, meeting clients’ needs must be the starting point for mental health care (5). Our findings indicate that the PIR model was able to substantially reduce client needs.

Qualitative studies on the PIR initiative undertaken previously have shown that the model of care benefited not only clients and carers but also health professionals. Clients stated that they felt valued, got a better understanding of their illness, felt empowered to better engage with services, and were encouraged to make decisions about their lives (50, 51). Health professionals stated that the model of care promoted a team approach to client care and prevented duplication of services (50). It allowed them to better understand the roles of other professionals, improve relationships between organizations, and facilitate interagency collaboration (32). The model is also shown to be cost-effective (52). The PIR model can therefore be considered a useful recovery-oriented model of care for persons with SPMI.

This is perhaps the first study that shows that higher-level safety and esteem needs tend to get met when accommodation needs, which is a basic physiological need, get met. Previous evidence appears to be mixed. Whereas some have indicated that a person’s higher-level needs usually come into play after basic needs are met (53), others argue that the hierarchy was more complicated (54). Nonetheless, MacPherson and colleagues suggest that services can more effectively address peoples’ needs when they have housing (55), although it was not clear what specific factors could have contributed to those findings.

The present study found that meeting accommodation needs significantly increased the likelihood of meeting needs related to money, childcare, food, safety to self, education, and access to services. It is likely that people who were homeless did not attend their compulsory social service appointments regularly and hence did not receive their fortnightly payments. Once clients had stable accommodation, they were usually taught how to manage money and were supported to budget their income for food and payment of rent. Having a secure home also enabled parents (particularly single mothers) to better look after their children.

When accommodation needs were met, clients were able to learn about day-to-day living skills, such as buying better food from the supermarket. People in a stable home could also be linked into voluntary organizations that provided food to the home. In addition, when clients lived close to a food distribution point, they could access it without assistance. Once accommodation needs were met, clients did not have to worry about their next meal or where they would sleep. It was therefore easier to assist them in developing an action plan that focused on what they needed to do next. This empowered them to think about issues, such as education and employment. Other authors have also reported that permanent housing did enable clients to consider subsequent goals to improve one’s life (54). When accommodation was located close to services that supported education, access was made easier. There are suggestions that meeting accommodation needs tends to show improvements in mental health problems as well, although the evidence is still not robust (56).

It is widely accepted that social determinants, such as housing and employment, have a significant bearing on the mental health of individuals, and providing social and other nonclinical services is essential for their well-being (57). Although in Australia, the National Disability Insurance Scheme (NDIS) has been given the resources to assist persons with SPMI, funding allocations are far below estimated requirements (57). Even so, there is optimism for the future because Australia is in the process of identifying areas for mental health reform through the Productivity Commission’s inquiry into mental health (58). Commissioned in November 2018, this 18-month inquiry will examine how sectors, such as education, employment, social services, housing, and justice, can contribute to improving mental health and economic participation of persons with mental illness (58).

The reasons for the relatively high dropout rate in this study are unclear. Previous reports suggest that associations with disengagements with mental health services are complex and encompass sociodemographic and clinical variables, as well as variables related to service provision (59). The PIR model is quite new and different from traditional mental health care models. Anecdotally, service providers presume that dropouts could have been caused by the exacerbation of symptoms or transfer to another program. Symptoms in SPMI are known to wax and wane. When symptoms become worse, many people (particularly young people) tend to temporarily disengage from services and return when they feel better. As a result, there can be several cases of dropouts and re-enrollments. The PIR initiative was a new and innovative program in Australia. When individuals who were used to a system that did not necessarily take care of their needs became involved with it, they are likely to have been empowered to make it work for them, thereby transferring to the program that was closer to family and other supports. As a result, dropouts in this initiative could also have included a new enrollment in a neighboring program.

There are a few limitations in this study. No data on recovery were available, although another group has reported improved recovery in participants enrolled in the PIR initiative (60). There was also no control group, and although its focus was to address unmet needs, it is difficult to attribute change in needs entirely to the initiative.

## Conclusion

There was a significant reduction in unmet needs reported by clients who enrolled in the care coordination model of the PIR initiative. The highest reductions in needs were for safety to others, cultural or spiritual, information on condition, benefits, and self-care. The least reductions in unmet needs were reported for psychological distress, company, drug abuse, childcare, and alcohol abuse. Meeting accommodation needs was associated with meeting needs related to money, childcare, food, safety to self, education, and access to other services. Care coordination is a useful way to address multiple and complex needs of persons with SPMI. While addressing needs, priority must be given to meeting accommodation needs.

## Data Availability

The data sets for this manuscript are not publicly available because of copyright issues. Requests to access the data sets should be directed to Gippsland PHN (info@gphn.org.au).

## Author Contributions

AI conceived the idea for the study and wrote the initial drafts. NK helped obtain the data for the study. AB undertook data analysis. KS and NK contributed to the development of the manuscript. All authors read and approved the final version of the manuscript.

## Funding

This study was funded by the Gippsland Primary HealthNetwork.

## Conflict of Interest Statement

NK works for the Gippsland Partners in Recovery initiative, which funded this study.

The remaining authors declare that the research was conducted in the absence of any commercial or financial relationships that could be construed as a potential conflict of interest.

## References

[1] Fleury M-J Grenier G Bamvita J-M Piat M Tremblay J Adequacy of help received among individuals with severe mental disorders. Adm Policy Ment Health Ment Health Serv Res (2014) 41(3):302–16. 10.1007/s10488-013-0466-8 23334467

[2] Ruggeri M Leese M Slade M Bonizzato P Fontecedro L Tansella M Demographic, clinical, social and service variables associated with higher needs for care in community psychiatric service patients. Soc Psychiatry Psychiatr Epidemiol (2004) 39(1):60–8. 10.1007/s00127-004-0705-0 15022048

[3] Fleury M-J Piat M Grenier G Bamvita J-M Boyer R Lesage A Components associated with adequacy of help for consumers with severe mental disorders. Adm Policy Ment Health Ment Health Serv Res (2010) 37(6):497–508. 10.1007/s10488-010-0292-1 20204492

[4] Drukker M van Dillen K Bak M Mengelers R Van Os J Delespaul P The use of the Camberwell Assessment of Need in treatment: what unmet needs can be met? Soc Psychiatry Psychiatr Epidemiol (2008) 43(5):410–7. 10.1007/s00127-007-0301-1 18163188

[5] Slade M Leese M Cahill S Thornicroft G Kuipers E Patient-rated mental health needs and quality of life improvement. Br J Psychiatry (2005) 187:256–61. 10.1192/bjp.187.3.256 16135863

[6] Lasalvia A Bonetto C Malchiodi F Salvi G Parabiaghi A Tansella M Listening to patients’ needs to improve their subjective quality of life. Psychol Med (2005) 35(11):1655–65. 10.1017/S0033291705005611 16219123

[7] Schiotz ML Host D Frolich A Involving patients with multimorbidity in service planning: perspectives on continuity and care coordination. J Comorb (2016) 6(2):95–102. 10.15256/joc.2016.6.81 29090180 PMC5556451

[8] Fleury M-J Grenier G Bamvita J-M Tremblay J Factors associated with needs of users with severe mental disorders. Psychiatr Q (2013) 84(3):363–79. 10.1007/s11126-012-9252-0 23224403

[9] Isaacs AN Beauchamp A Sutton K Maybery D Unmet needs of persons with a severe and persistent mental illness and their relationship to unmet accommodation needs. Health Soc Care Community (2019) 27(4):e246–e256. 10.1111/hsc.12729 30848020

[10] Wiersma D Needs of people with severe mental illness. Acta Psychiatr Scand Suppl (2006) 429(suppl): 115–9. 10.1111/j.1600-0447.2005.00728.x 16445493

[11] Toro PA Bellavia CW Daeschler CV Owens BJ Wall DD Passero JM Distinguishing homelessness from poverty: a comparative study. J Consult Clin Psychol (1995) 63(2):280–9. 10.1037/0022-006X.63.2.280 7751489

[12] Forchuk C Dickins K Corring DJ Social determinants of health: housing and income. Healthc Q (Toronto, Ont). (2016) 18:27–31. 10.12927/hcq.2016.24479 26854545

[13] Forchuk C Turner K Joplin L Schofield R Csiernik R Gorlick C Housing, income support and mental health: Points of disconnection. Health Res Policy Syst (2007) 5, 14. 10.1186/1478-4505-5-14 18072980 PMC2238740

[14] Australian Institute of Health and Welfare Specialist homelessness services:2012–2013. Cat. no. HOU 27. Canberra: Australian Institute of Health and Welfare (2013).

[15] O’Campo P Hwang SW Gozdzik A Schuler A Kaufman-Shriqui V Poremski D Food security among individuals experiencing homelessness and mental illness in the At Home/Chez Soi Trial. Public Health Nutr (2017) 20(11):2023–33. 10.1017/S1368980017000489 PMC10261387 28560947

[16] Lee BA Greif MJ Homelessness and hunger. J Health Soc Behav (2008) 49(1):3–19. 10.1177/002214650804900102 18418982 PMC4121392

[17] Baggett TP Singer DE Rao SR O’Connell JJ Bharel Monica Rigotti N A Food insufficiency and health services utilization in a national sample of homeless adults. J Gen Intern Med (2011) 26(6):627–34. 10.1007/s11606-011-1638-4 PMC3101971 21279455

[18] Herault N Ribar DC Food insecurity and homelessness in the Journeys Home survey. J Hous Econ (2017) 37(37):52–66. 10.1016/j.jhe.2017.05.001

[19] Jocoy CL Del Casino VJ Jr. Homelessness, travel behavior, and the politics of transportation mobilities in Long Beach, California. Environ Plan A (2010) 42(8):1943–63. 10.1068/a42341

[20] Hodnicki DR Horner SD Homeless Mothers’ Caring for Children in a shelter. Issues Ment Health Nurs (1993) 14(4):349–56. 10.3109/01612849309006898 8244687

[21] Crowe C Hardill K Nursing research and political change: the street health report. Can Nurse (1993) 89(1):21–4. 8425165

[22] Pizem P Massicotte P Vincent JR Barolet RY The state of oral and dental health of the homeless and vagrant population of Montreal. J Can Dent Assoc (1994) 60(12):1061–5. 7842371

[23] Wrenn K Foot problems in homeless persons. Ann Intern Med (1990) 113(8):567–9. 10.7326/0003-4819-113-8-567 2205141

[24] Wrenn K Immersion foot. A problem of the homeless in the 1990s. Arch Intern Med (1991) 151(4):785–8. 10.1001/archinte.151.4.785 2012466

[25] Hwang SW Homelessness and health. Can Med Assoc J (2001) 164(2):229–33. PMC80688 11332321

[26] Gelberg L Linn LS Assessing the physical health of homeless adults. JAMA (1989) 262(14):1973–9. 10.1001/jama.262.14.1973 2778933

[27] Bhui K Shanahan L Harding G Homelessness and mental illness: a literature review and a qualitative study of perceptions of the adequacy of care. Int J Soc Psychiatry (2006) 52(2):152–65. 10.1177/0020764006062096 16615247

[28] Kerman N Sylvestre J Polillo A The study of service use among homeless persons with mental illness: a methodological review. Health Serv Outcomes Res Methodol (2016) 16(1-2):41–57. 10.1007/s10742-016-0147-7

[29] Whiteford H McKeon G Harris M Dimini S Siskin D Scheurer R System-level intersectoral linkages between the mental health and non-clinical support sectors: a qualitative systematic review. Aust N Z J Psychiatry (2014) 48(10):895– 906. 10.1177/0004867414541683 25002710

[30] Randolph F Blasinsky M Leginski W Parker LB Goldman HH Creating integrated service systems for homeless persons with mental illness: the ACCESS Program. Access to Community Care and Effective Services and Supports. Psychiatr Serv (1997) 48(3):369–73. 10.1176/ps.48.3.369 9057240

[31] Lee S Ad Castella J Kennedy A Kroschel J Humphrey C Kerr R Mental health care on the streets: An integrated approach. Aust N Z J Psychiatry (2010) 44(6):505–12. 10.3109/00048670903555120 20482410

[32] Isaacs AN Firdous F A care coordination model can facilitate interagency collaboration when designing recovery-oriented services. J Psychosoc Nurs Ment Health Serv (2019) 57(5):38–43. 10.3928/02793695-20181128-01 30508461

[33] Hannigan B Simpson A Coffey M Barlow S Jones A Care coordination as imagined, care coordination as done: findings from a cross-national mental health systems study. Int J Integr Care (2018) 18(3):12. 10.5334/ijic.3978 PMC6137622 30220895

[34] Bowers A Owen R Heller T Care coordination experiences of people with disabilities enrolled in medicaid managed care. Disabil Rehabil (2017) 39(21):2207–14. 10.1080/09638288.2016.1219773 27548093

[35] Sutton K Isaacs AN Dalziel K Maybery D Roles and competencies of the Support Facilitator in Australia. Aust Health Rev (2017) 41:91–7. 10.1071/AH15183 27074116

[36] Australian Government Department of Health 2015 About Partners in Recovery.” Australian Government, accessed 21st January http://www.health.gov.au/internet/main/publishing.nsf/Content/mental-pir-about.

[37] Australian Government Department of Health Partners in Recovery: coordinated support and flexible funding for people with severe and persistent mental illness with complex needs (PIR).” Australian Government, accessed 21st January 2016 http://www.health.gov.au/internet/main/publishing.nsf/Content/mental-pir.

[38] Tay L Diener E Needs and subjective well-being around the world. J Pers Soc Psychol (2011) 101(2):354–65. 10.1037/a0023779 21688922

[39] Maslow AH Theory of human motivation. Psychol Rev (1943) 50(4):370–96. 10.1037/h0054346

[40] McLeod SA Maslow's hierarchy of needs. Simply Psychology. (2018). Retrieved from www.simplypsychology.org/maslow.html

[41] Maslow AH Motivation and personality. New York: Harper and Row (1954).

[42] Regional Development Victoria Gippsland Regional Plan 2015-2020. edited by Regional Development Victoria. Melbourne: Victorian Government (2015).

[43] Fixus technologies 2014 “Fixus.” accessed 21st July http://fixus.com.au/.

[44] Evans S Greenhalgh J Connelly J Selecting a mental health needs assessment scale: guidance on the critical appraisal of standardized measures. J Eval Clin Pract (2000) 6(4):379–93. 10.1046/j.1365-2753.2000.00269.x 11133121

[45] Wennstrom E Sorbom D Wiesel FA Factor structure in the Camberwell Assessment of Need. Br J Psychiatry (2004) 185:505–10. 10.1192/bjp.185.6.505 15572742

[46] Slade M CAN: Camberwell assessment of need: a comprehensive needs assessment tool for people with severe mental illness. London: Gaskell (1999).

[47] Australian Government Department of Health Partners In Recovery (PIR): PIR Client Minimum Data Set Ver.1.3. Canberra: Australian Government (2014).

[48] Stata Statistical Software: Release 15. StataCorp College Station, TX.

[49] Ochocka J Nelson G Janzen R Moving forward: negotiating self and external circumstances in recovery. Psychiatr Rehabil J (2005) 28:315–22. 10.2975/28.2005.315.322 15895914

[50] Isaacs AN Sutton K Dalziel K Maybery D Outcomes of a care coordinated service model for persons with severe and persistent mental illness: a qualitative study. Int J Soc Psychiatry (2017) 63(1):40–7. 10.1177/0020764016678014 28135998

[51] Waks S Scanlan JN Berry B Schweizer R Hancock N Honey A Outcomes identified and prioritised by consumers of Partners in Recovery: a consumer-led study. BMC Psychiatry (2017) 17(1):338. 10.1186/s12888-017-1498-5 28985728 PMC6389213

[52] Isaacs AN Dalziel K Sutton K Maybery D Referral patterns and implementation costs of the Partners in Recovery initiative in Gippsland: learnings for the National Disability Insurance Scheme. Aust Psychiatry (2018) 26(6):586–589. 10.1177/1039856218759408 29457488

[53] Nelson G Aubry T Lafrance A A review of the literature on the effectiveness of housing and support, assertive community treatment, and intensive case management interventions for persons with mental illness who have been homeless. Am J Orthopsychiatry (2007) 77(3):350–61. 10.1037/0002-9432.77.3.350 17696663

[54] Henwood BF Derejko KS Couture J Padgett DK Maslow and mental health recovery: a comparative study of homeless programs for adults with serious mental illness. Adm Policy Ment Health (2015) 42(2):220–8. 10.1007/s10488-014-0542-8 PMC4130906 24518968

[55] MacPherson R Gregory N Slade M Foy C Factors associated with changing patient needs in an assertive outreach team. Int J Soc Psychiatry (2007) 53(5):389–96. 10.1177/0020764007078338 18018661

[56] Enns JE Holmqvist M Wener P Rothney J Halas G Kosowan L Interventions aimed at reducing poverty for primary prevention of mental illness: a scoping early review. Ment Health Prev (2019) 15. 10.1016/j.mhp.2019.200165

[57] Rosenberg S Shangri-La and the integration of mental health care in Australia. Public Health Res Pract (2017) 27(3):e2731723. 10.17061/phrp2731723 28765856

[58] Frydenberg J Hunt G 2018 Productivity Commission inquiry into mental health terms of reference. Hunt, G., accessed 30th April http://www.health.gov.au/internet/ministers/publishing.nsf/Content/02F85D7E06982F82CA25834E00063F60/$File/GH158.pdf.

[59] O’Brien A Fahmy R Singh SP Disengagement from mental health services. A literature review. Soc Psychiatry Psychiatr Epidemiol (2009) 44(7):558–68. 10.1007/s00127-008-0476-0 19037573

[60] Hancock N Scanlan JN Gillespie JA Smith-Merry J Yen I Partners in Recovery program evaluation: changes in unmet needs and recovery. Aust Health Rev (2018) 42(4):445–52. 10.1071/ah17004 28693718

